# Lifestyle and Host Defense Mechanisms of the Dung Beetle, *Euoniticellus intermedius*: The Toll Signaling Pathway

**DOI:** 10.1673/031.013.10801

**Published:** 2013-10-22

**Authors:** Rodney Hull, Mohamed Alaouna, Lucky Khanyile, Marcus Byrne, Monde Ntwasa

**Affiliations:** 1 School of Molecular & Cell Biology. University of the Witwatersrand, Johannesburg. Private Bag X3, Wits. 2050, South Africa; 2 School of Animal and Plant Sciences. University of the Witwatersrand, Johannesburg. Private Bag X3, Wits. 2050, South Africa

**Keywords:** antimicrobial peptides, beetle immunity, innate immunity

## Abstract

The dung beetle, *Euoniticellus intermedius* (Reiche) (Coleoptera: Scarabaeidae) is an important ecological and agricultural agent. Their main activity, the burying of dung, improves quality of the soil and reduces pests that could cause illness in animals. *E. intermedius* are therefore important for agriculture and for good maintenance of the environment, and are regarded as effective biological control agents for parasites of the gastrointestinal tract in livestock. The ability of *E. intermedius* to co-exist comfortably with many microorganisms, some of which are important human pathogens, stimulated our interest in its host defense strategies. The aim of this study was to investigate the Toll signaling pathway, which is strongly activated by fungi. Gene expression associated with fungal infection was analyzed by using 2-D gel electrophoresis and mass spectroscopy. Furthermore, the partial adult transcriptome was investigated for the presence of known immune response genes by using high-throughput sequencing and bioinformatics. The results presented here suggest that *E. intermedius* responds to fungal challenge via the Toll signaling pathway.

## Introduction

The dung beetle, *Euoniticellus intermedius* (Reiche) (Coleoptera: Scarabaeidae), belongs to the most diverse order of insects, with more described species than any other in the animal and plant kingdoms (Farrell 1998). The two main branches of Class Insecta are the hemimetabolous insects, such as the grasshopper, which have incomplete metamorphosis, and the holometabolous insects, such as the beetles, which have complete metamorphosis. These branches split about 416 m.y.a. (Labandeira and Phillips 1996; Hoffmann 2004; Erezyilmaz 2006).

*Drosophila melanogaster* Meigen (Diptera: Drosophilidae) is another holometabolous insect and is regarded as the most geneticallytractable and widely-studied laboratory model of the holometabolous insects, but there is evidence that it is highly specialized and does not fully represent invertebrate evolutionary characteristics. For example, the beetle *Tribolium castaneum* (Herbst) (Coleoptera: Tenebrionidae) shares with vertebrates ancestral genes that are not present in *D. melanogaster*. For example, components of some signaling pathways that are conserved in coleopterans and vertebrates are not conserved in *D. melanogaster* (Van der Zee et al. 2008). Moreover, *T. castaneum* embryogenesis has retained the ancestral short-germ band mode, which resembles vertebrates (Tautz and Sommer 1995; Handel et al. 2000; Liu and Kaufman 2005). A recent study on protein evolution suggests that coleopterans have lower rates of divergence when compared to dipterans where a highly accelerated protein evolution is evident (Savard et al. 2006). Coleopterans are therefore likely to be more suitable for comparative studies against vertebrates than the dipterans.

Due to their important agricultural benefits, *E.intermedius* are often introduced to control ecological damage. Some of the benefits include nutrient recycling, improvements to soil tilth, and pest control (Bertone et al. 2006). Tunneling beetles, such as *E. intermedius*, are beneficial to pasture health, as they enhance soil conditions by increasing percolation. Furthermore, dung beetles reduce the number of parasites acquired by cattle (Fincher 1975). They also reduce the population of pestiferous flies, such as the African buffalo fly (*Haematobia thirouxi potans* (Bezzi)), the Australian buffalo fly (*Haematobia irritans exigua* de Meijere), the bush fly (*Musca vetustissima* Walker), the face fly (*Musca autumnalis* De Geer), and the horn fly (*Haematobia irritans irritans* (L.)) (Lastro 2006). Consequently, the introduction of *E. intermedius* into new habitats has a high impact on the environment. *E.intermedius* was introduced in Texas, USA, to control dung accumulation, and the beetles spread quickly to Mexico, where they had a positive impact on soil fertility and productivity. *E. intermedius* also help control nematodes that are potential cattle parasites (Montes and Halffter 1998; Anduaga 2004).

Adult *E. intermedius* feed on the microbe-rich particulate portion of the dung and do not actually consume the dung fibers (Holter and Scholtz 2007). The larvae, on the other hand, consume most of the dung fibers in the brood balls. Development from egg to adult takes 5 to 6 weeks, and adults have a lifespan of about 2 months. The evolutionary history and lifestyle of dung beetles point to an interesting immune system that could be more comparable to vertebrate innate immunity than *D.melanogaster* in some respects.

The general trend in insect immune signaling is that recognition of pathogens depends upon the existence of molecular patterns, called pathogen associated molecular patterns, on the cell wall of pathogens and the pattern recognition receptors in the immune system of the target organism. Lysine containing peptidoglycans found in Gram-positive bacteria and b-1,3-glucans in fungi stimulate the Toll signaling pathway, while diaminopimelic acid containing peptidoglycans found in Gramnegative bacteria and some Gram-positive bacilli stimulate the immune deficiency pathway (Hoffmann 2004; Zou et al. 2007; Altincicek et al. 2008; Valanne et al. 2011). In *D. melanogaster*, it has been shown that virulence factors and pathogen associated molecular patterns in fungi and bacteria activate the Toll pathway via two parallel protease cascades that culminate in the cleavage of pro-spaёtzle to produce the active physiological ligand of Toll. The virulence factors, such as cell wall components, in live organisms activate the Toll pathway via pattern recognition receptors and a serine protease cascade, which eventually cleaves the inactivate pro-spaёtzle to release an active C-terminal fragment (C-106), which then binds to Toll. The parallel pathway is induced by pathogen-secreted proteases and proceeds via a cascade involving Persephone (Psh) and activation of Toll by activated spaёtzle (Imler et al. 2004; Chamy et al. 2008; Kellenberger et al. 2011; Valanne et al. 2011). Intracellular signals transduced by the binding of spaёtzle and Toll are relayed by a phosphorylation cascade that culminates in the cytoplasmic release and nuclear localization of NFkB-like transcription factors. The Toll pathway leads to the release of the dorsal immune factor transcription factor, which activates the expression of various effector genes, primarily antimicrobial peptides.

In recent years, ground-breaking work in coleopteran immunity has been accumulating, and the nature of the signaling network is emerging. Studies using the larger beetles, *Tenebrio molitor* and *Holotrichia diomphalia*, show that beetles responded to b-1,3-glucans and to lysine containing peptidoglycan and polymeric diaminopimelic acid containing peptidoglycans by using the Toll signaling pathway. Both types of pathogen associated molecular patterns form complexes with Gram-negative binding protein 3(GNBP3) in fungi and peptidoglycan recognition protein in Gram-negative bacteria. These complexes activate the serine protease cascade via an apical protease known as the modular serine protease, followed by two types of CLIP domain serine proteases at penultimate and terminal positions before pro-spaёtzle (Park et al. 2010; Kellenberger et al. 2011; Ntwasa et al. 2012). The studies on beetles revealed important pieces of evidence indicating that beetle immunity might differ from that of flies. For example, studies conducted on the red flour beetle revealed that the peptidoglycan recognition proteins, used by invertebrates to sense pathogens, diversified in beetles before the radiation of holometabolous insects, while in *D. melanogaster* there has been sustained diversification through numerous duplications. Furthermore, many immune related genes, including the CLIP-domain serine proteases and their inhibitors, Toll related proteins, and antimicrobial peptides, diversified extensively through evolution most likely because of the diverse habitats of the species (Zou et al. 2007).

There have been no significant molecular studies conducted on *E. intermedius* thus far, except for those involving the relationship between prophenoloxidase and immunity (Pomfret and Knell 2006). The aim of this study was to assess the immune defense pathways that are activated when *E. intermedius* is infected by fungi. It is part of a broader investigation of host defense mechanisms adopted by the dung beetle. The life cycle of *E. inter medius* is presented to highlight that it is dominated by microbes, some of which are human pathogens. The larval and adult stages are particularly dependent on these microbes for nutrition. Proteomic and bioinformatics analysis of the adult transcriptome of the dung beetle indicates that the Toll signaling pathway may be conserved and probably activated by fungi.

## Materials and Methods

### Rearing beetles and microscopy

Beetles were bred in 160 × 130 × 130 mm plastic containers, which were half full with soil. Cow dung was placed on the soil, and 3 breeding pairs of beetles (3 males and 3 females) were added to the containers. Every 3 to 4 days, frozen and then thawed cow dung was placed in the container. Once a week, the soil was sieved, and brood balls were removed. If the breeding pair had survived, they were moved to a new container with fresh soil, and dung and the brood balls were placed in a large (400 × 300 × 200 mm) plastic container covered with compacted soil. A wet sponge was then placed on the soil to keep it moist. Once beetles began to emerge, small, plastic dishes baited with dung were used as traps to capture them within the bins. These beetles were used as new breeding pairs or in experimental work.

### Treatment of beetles

Adult male and female beetles were infected with the entomopathogenic fungus *Beauveria bassiana* (strain 80.2), and samples of haemolymph were collected before and after infection. Ten beetles were placed on a 90 mm plate containing *B. bassiana* (i.e., 1 beetle per 6.36 cm^2^). The plate was then placed on a shaker for 5 minutes, after which the beetles were transferred to a small jar containing sterile soil and dung and then left overnight to allow an immune response to occur.

### Collection and preparation of haemolymph samples

To collect haemolymph from adult beetles, the exoskeleton was pierced below the hind limb using a heated tungsten spike. A 10 ml microsyringe needle was then pushed through this hole. The plunger was slowly withdrawn, and between 2 and 5 µL of haemolymph was collected and pooled from 5 beetles without discriminating between genders. The extracted haemolymph was diluted 1:4 in extraction buffer (0.1% trifluoroacetic acid (to obtain pH3), 10 µg/mL aprotinin, 1 µM leupeptin, 15 µM phenylmethylsulfonyl fluoride, and 20 µM phenylthiourea). The samples were not centrifuged to remove hemocytes, because both extracellular and intracellular events at this stage were of interest. Protein concentration was measured by the Bradford method (Bradford 1976).

### 2-dimensional gel electrophoresis

Haemolymph proteins were re-suspended in 2-D PAGE re-hydration buffer (8 M urea, 2% CHAPS, 50 mM DTT, 0.2% Bio-lyte, 0.5 % Bromophenol Blue) and applied onto 7 cm immobilized pH 3–10 gradient strips (Bio-Rad, www.bio-rad.com). Passive rehydration was allowed to occur for 12 hours at 20° C. Isoelectric focusing was performed in the Protean IEF system (Bio-Rad) according to the following program: an initial low voltage (250 V) 20-minute linear ramping step, followed by a high voltage (4000 V) 2-hour linear ramping step. The final step was a rapid ramping step for 10,000 volt hours. The strip was equilibrated in a new rehydration tray and covered with equilibration solution one (6 M urea, 2% SDS, 0.375 M Tris HCl pH 8.8, 20% glycerol, 2% DTT). After shaking for 10 minutes, equilibration solution one was replaced with equilibration solution two (6 M urea, 2% SDS, 0.375 M Tris HCl pH 8.8, 20% glycerol, 2.5% iodoacetamide) and shaken for 10 more minutes. The strips were then briefly placed in SDS running buffer and placed on a 10% polyacrylamide separating gel (Laemmli 1970), and then overlaid with agarose. The 2-D gel was then run for 40 minutes at 200 V and subsequently stained with Coomasie blue.

### Image Analysis

The two gels representing samples from treated and untreated beetles were digitized using a GS800 calibrated densitometer (Bio-Rad), and the resulting images were analyzed using the PDQuest 2-D Analysis Software Version 6.2 (Bio-Rad). Spot detection was carried out with a sensitivity setting of 102.21, using the treated sample gel as the master gel. 464 and 374 spots were respectively detected in the treated and untreated sample gel images. The analysis software was used to select spots for identification by mass spectrometry. Many spots within the Mr range 10–100 kDa and the *p*I range 4–7, based on whether they were downor up-regulated, were selected.

Spots were initially matched using the automated matching function of the program. Extended matching was then performed using the classical matching function. Both these methods involved matching spots based on the position of landmark spots. Manual spot matching and analysis was then performed across the gel images. This resulted in a final spot count of 135 spots in the master gel image, and 114 and 84 in the treated and the untreated images respectively.

Normalization was performed automatically by the program and was based on the total quantity in all valid spots option. This method assumed that changes in density average-out across the gels being analyzed.

The normalization formula used is as follows:

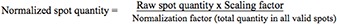
The scaling factor used was 10^6^ parts per million.

### Statistical analysis

Three biological replicates of haemolymph extracted from immune-challenged and unchallenged adult beetles were analyzed using 2D-PAGE and the PDQuest software. The means of density values from these biological replicates were used to construct the graphical representations of the optical density values for each spot. Two-sample *t*tests were performed to test for statistically significant differences between the challenged and unchallenged groups for each spot at the 95% confidence interval. Two *t*-tests were computed, one assuming equal group variances and the other assuming different group variances. An F-test for the null hypothesis that the immune-challenged and unchallenged populations have the same variance was also performed. The results of these tests were presented as *p-* and F-values. All statistical tests were performed using the Statistix 8 software package (www.statistix.com).

### Matrix laser desorption-time-of-flight mass spectrometry

Selected spots were manually excised from the gels and placed in a 5% acetic acid solution. After tryptic in-gel digestion following the method of Rabilloud (Rabilloud et al. 2001), the proteins were analyzed by mass spectroscopy either the matrix laser desorption-time-of-flight or the NanoLC-MS/MS (Bflex III Brüker Daltonics, a CapLC capillary LC system (Waters, www.waters.com) coupled to a hybrid quadrupole orthogonal acceleration time-of-flight tandem mass spec trometer (Q-TOF Micro, Waters). The sample (5 µl) was first concentrated and cleaned in a C18 PepMap precolumn cartridge (LC Packings/Dionex, www.dionex.com) and then separated on-line by the analytical reversedphase capillary column (Pepmap C18, 75 µm inner diameter, 15 cm length; LC Packings) under a 200 nL min^-1^ flow rate. The gradient profile used consisted of a linear gradient from 97% A (97.9% H_2_O; 2% ACN, 0.1% (v/v) HCOOH) to 95% B (98% ACN, 1.9% H_2_O, 0.1% (v/v) HCOOH) in 45 minutes, followed by a linear gradient to 95% B in 3 minutes.

The spray system (liquid junction) was used at 3.6 kV. Mass data acquisitions were piloted by MassLynx 4.0 software (Waters).NanoLCMS/ MS data were collected by datadependent scanning, that is, automated MS to MS/MS switching. Fragmentation was performed using argon as the collision gas, and with a collision energy profile optimized for various mass ranges of ion precursors. Four ion precursors were allowed to be fragmented at a time. Mass data collected during a NanoLC-MS/MS analysis were processed and then submitted to *de novo* sequencing. Peak lists were generated by ProteinLynx 2.1 software (Waters) with internal lockspray calibration (leucine enkephalin at 556.2771 m/z). There was no smoothing. Fragmentation spectra were loaded onto the Peptide Sequencing software (BioLynx, Waters) and the sequences were processed manually before being submitted to a BLAST search without any taxonomy restriction.

### Searching the *Euoniticellus intermedius* database for immune genes and other bioinformatics tools

The *E. intermedius* database contains a partial transcriptome of the untreated adult beetle (Khanyile et al. 2008). Sequences of innate immune response proteins were obtained from the UniprotKB/Swiss-Prot database using gene ontology identity number and species name. Protein sequences were selected from the human, *D. melanogaster* and *T. castaneum* databases. These included 600 human, 6000 *Drosophila* and 12 *Tribolium* sequences, and were deposited in a MySQL local database. Using TBLASTX, the local database was compared with the *E. intermedius* unigene database at a cut-off e-value of 1e-5.

## Results

### *Euoniticellus intermedius* life cycle in microbe-rich environment

**Figure 1.**
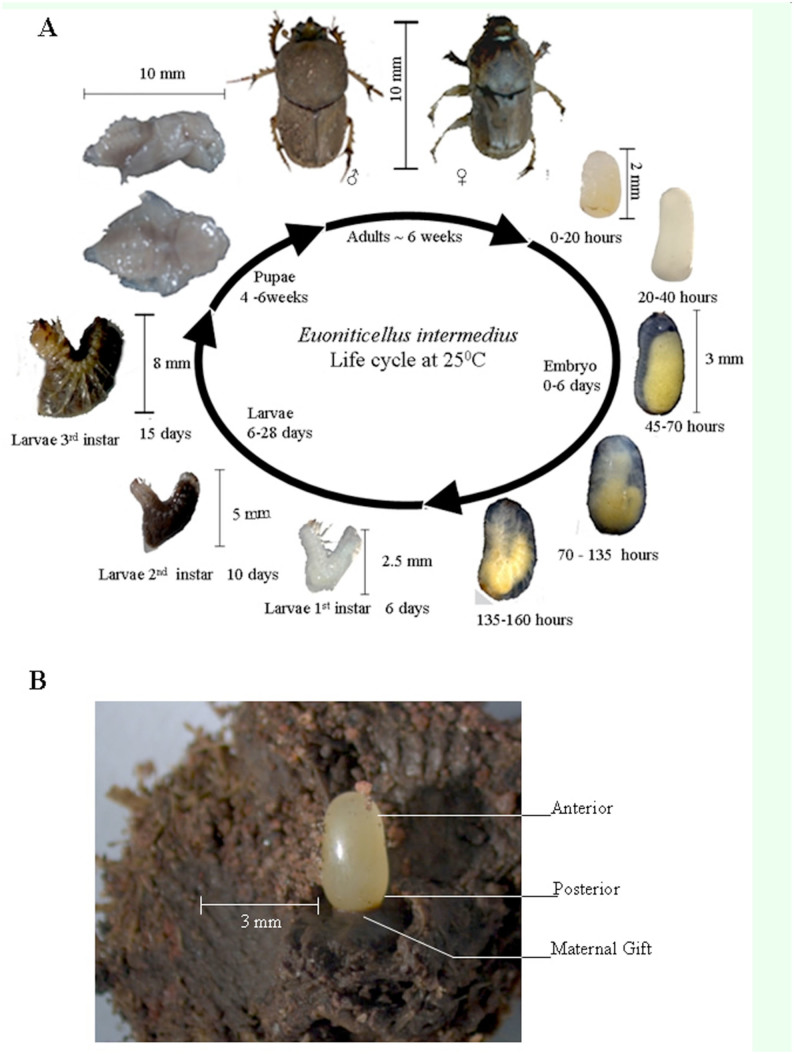
The life cycle of *Euonoticellus intermedius*. A. Developmental stages from embryo to adult. B. An embryo in a brood ball showing the maternal gift. High quality figures are available online.

The entire life cycle of *E. intermedius* occurs in cow dung (Figure 1A). Their adaptation to a habitat that is rich in different types of microbes, including human pathogens, suggests that dung beetles possess robust host-defense mechanisms. Extensive studies on the development of coleopterans have been conducted on *T. castaneum*, a short germ insect, but little is known about the developmental biology of *E. intermedius*.

The embryo develops in the brood ball apparently without physical contact with the interior surface. It is anchored on its posterior end ostensibly by the sticky black material termed the “maternal gift,” as it is placed there by the mother (Figure 1B). The role of the maternal gift has not been proven, but apart from acting as an anchor for the embryo, it could serve other purposes (Byrne et al. 2013). The features of the developing larvae are typical of other scarab beetles; with a dorsal expansion (or “hump”) at the middle of the body and caudal flattening (Edmonds and Halffter 1978). This seems to be a physical adaptation to facilitate movement of the larvae in the confines of the brood ball as it rotates within the ball to feed on dung. Feeding seems to start at the second larval instar, as these larvae look white and free of dung before this stage. At later stages, the gut system resembles a “bag of dung.”

### *Euoniticellus intermedius* immune response to fungal challenge

**Figure 2.**
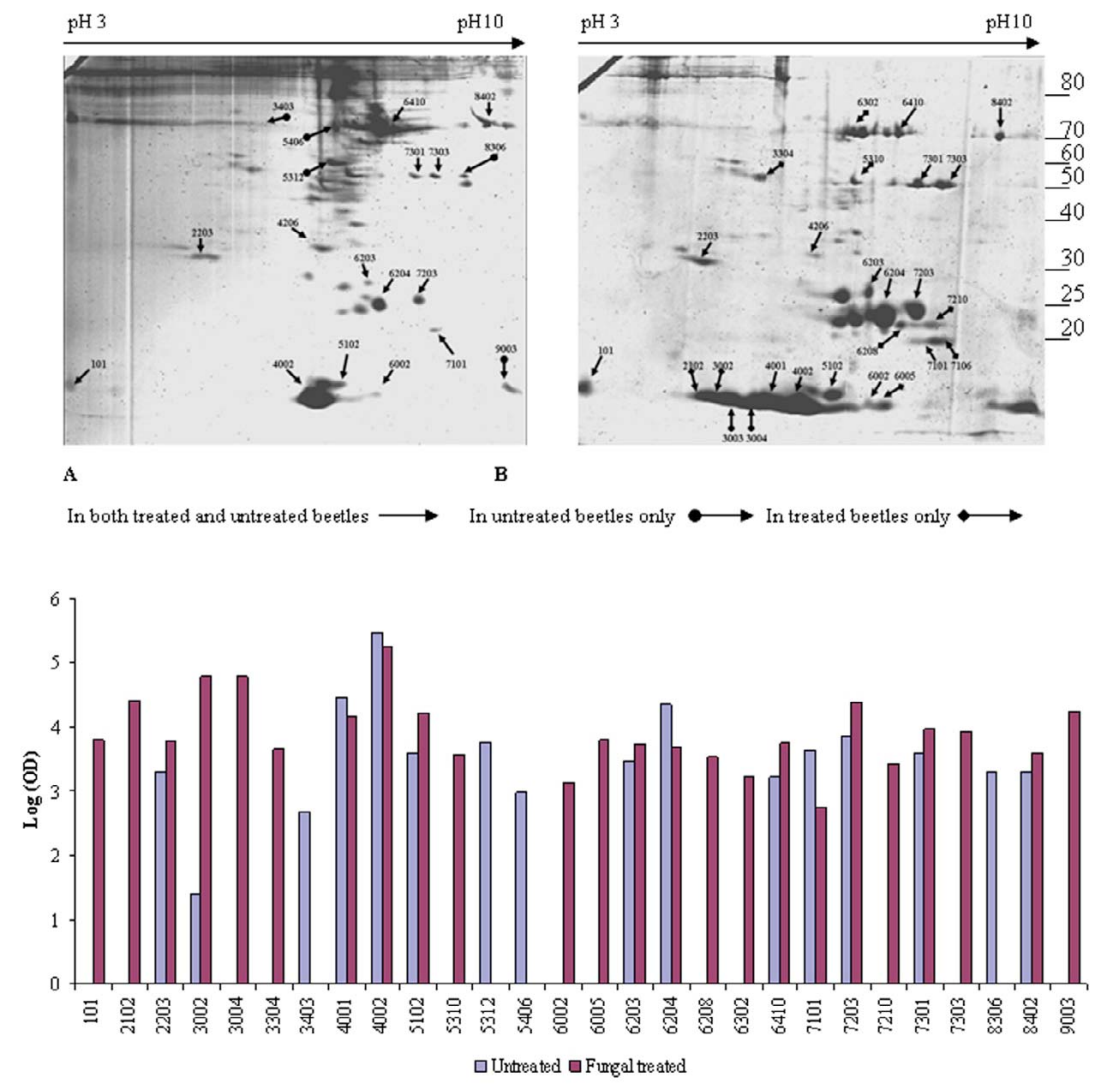
2-D gel electrophoresis of *Euonoticellus intermedius* hemolymph proteins*.* The gel was stained with Coomasie blue. It shows protein spots in unchallenged (A) and in infected beetles (B). Proteins found in treated beetles only, in untreated beetles only, and in both treated and untreated beetles are shown. C. Optical density measurements (N= 3) of protein spots. High quality figures are available online.

2-D gel electrophoresis of haemolymph from adult beetles before and after infection with the entomopathogenic *B. bassiana* revealed differential expression of certain genes in response to fungal infection (Figure 2). Differentially expressed genes were determined by the PDQuest software in three separate experiments.

Initially, matrix laser desorption-time-of-flight was used to identify samples after in-gel tryptic digestion. The mass list obtained at this stage was used to search the United States National Center for Biotechnology Information (NCBI) database (www.ncbi.nlm.nih.gov) without any taxonomy restrictions using the Mascot search engine. None of the proteins could be identified by this method. Nano LC-MS/MS analysis was then performed in order to obtain sequence data. Short sequence fragments were then determined by *de novo* sequencing. BLAST search was performed on the NCBI BLASTP 2.2.14 on all non-redundant GenBank CDS containing, at this time, 3695564 entries. For many of the positively identified proteins, the MS-BLAST search engine was employed using the short peptide fragments for a given protein sequence that was used as input data into the MS BLAST search engine (http://dove.emblheidelberg.de/Blast2/msblast.html).

Proteins identified by MS-BLAST were then subjected to manual comparison with the *E. intermedius* database (http://flylab.wits.ac.za/EI/est2uni/blast.php) using TBLASTN (Table 1). In some cases, alternative peptides matched sequences in the same contig or singleton. For example, spots 2203 and 3304, which were up-regulated upon fungal infection, were predicted as serine proteases from the short amino acid sequences produced by *de novo* sequencing using Nano LC-MS/MS. Alternative peptides produced from these spots could be assigned to different parts of the same contig. Sequences of two peptide fragments associated with spot 2203 (with molecular weight of approximately 30 kDa) matched parts of the predicted CL8Contig1 protein sequence. Two of these fragments had 100% identity with peptide sequences in the CL8Contig1-encoded polypeptide, which is predicted to be a chymotrypsin- type serine protease like Psh or sphinx1/2. The peptide fragments obtained from spot 3304, at approximately 50 kDa, facilitated the identification of two contigs (CL20Contig1 and CL23Contig1) in the *E. intermedius* database using TBLASTN. These contigs are also predicted to encode a serine protease like Psh or sphinx. Correlation of these spots with Psh was consistent with response to fungal challenge as observed in *D.melanogaster* (Imler et al. 2004). Based on predicted molecular weights of *D. melanogaster* sphinx1/2 (25 kDa) and Psh (43–50 kDa, sphinx could be assigned to spot 2203 and Psh to spot 3304, but a definite identification will require full length sequencing and more biochemical analysis.

### Bioinformatics analysis of immune genes in the *Euoniticellus intermedius* transcriptome database

Comparison of the *E. intermedius* transcriptome database with databases of holometabolous (*A. melifera*, *B. mori*, *D. melanogaster*, and *T. castaneum*) and hemimetabolous (*A. pisum*) insects showed that *E. intermedius* was more comparable to *T. castaneum* than it is to other insect species (Table 2). Approximately 37% of the *E. in termedius* transcripts matched orthologues in the *D. melanogaster* database, indicating that the *D. melanogaster* database may be inadequate for functional annotation of the dung beetle genome. This apparent divergence could be due to evolutionary factors, as the primary radiation of coleopterans and dipterans occurred approximately 284 m.y.a., and dipterans have been shown to exhibit an accelerated rate of protein evolution (Savard et al. 2006).

**Figure 3.**
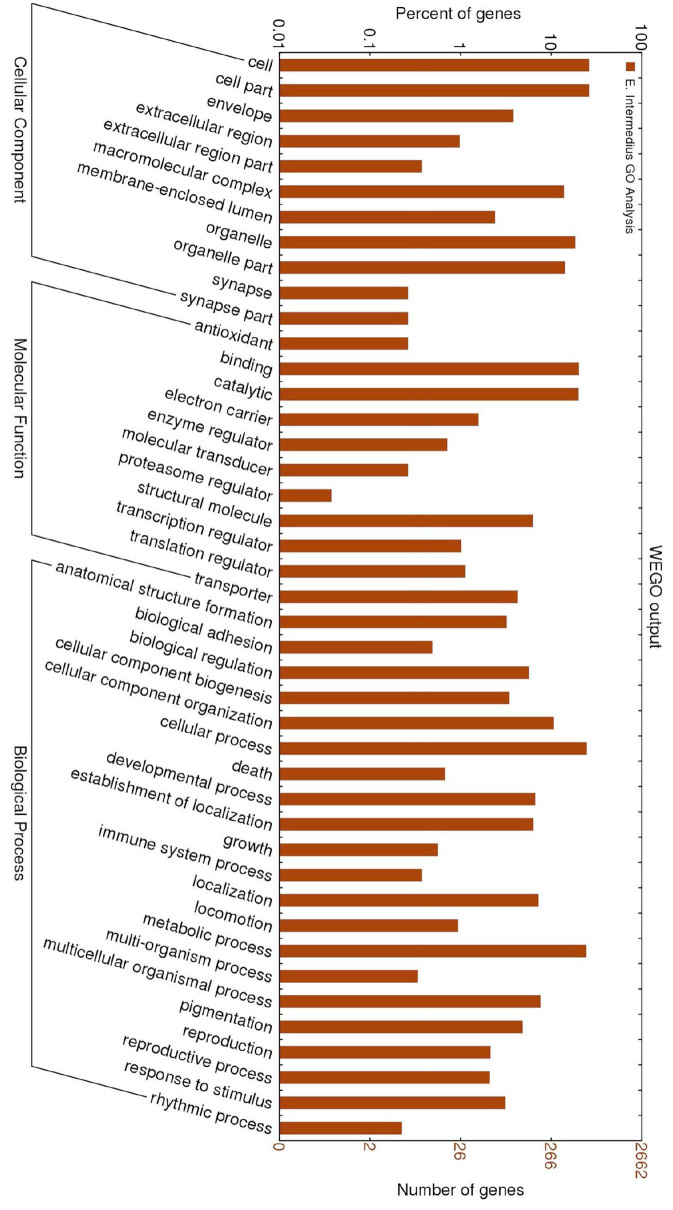
Gene Ontology Analysis of *Euonoticellus intermedius* genes. From the total of 2662 unigenes, 933 were functionally annotated by blast result using *Drosophila melanogaster*. A gene ontology study was then conducted on the 933 unigenes made up of 338 contigs and 595 singletons. The *D. melanogaster* gene ontology association file was used to annotate the unigenes with Gene Ontology terms, and results were stored in a local Mysql database. The gene ontology analysis is implemented by one of the modules of the EST2uni pipeline (Forment et al. 2008). The retrieval of all 2662 unigenes, left-joined by gene ontology terms, resulted in a WEGO (Web Gene Ontology) annotation plot. The Native Format file and WEGO were used for plotting GO annotation results as a histogram. The gene ontology terms have 1 to 7 levels of detail, 1 being most general and 7 most detailed/specific. The histogram shown here is of GO terms at level 2. High quality figures are available online.

Functional annotation against the *D. melanogaster* database revealed proteins involved in a range of biological processes and molecular functions (Figure 3). Since this ontological analysis provided general information about response to stress and did not specify immune-related genes, targeted analysis was conducted using more databases, as described in materials and methods. Consequently, more immune genes were predicted through comparison with genes in the human, *T. castaneum*, and *D. melanogaster* databases (Table 3).

Using this approach, several molecules associated with the Toll signaling pathway were predicted. CL8Contig1, CL20Contig1, CL15Contig1, and CL23Contig1 were predicted as either Psh or sphinx1/2. Others were predicted to be Toll-related genes (CL426Contig1, CL673Contig1) and spaёtzle (CL283Contig1). Manual searches using *Drosophila* or *Tribolium* sequences revealed another putative spaёtzle-encoding contig (Cl47contig1), a singleton (007956_1645_0789_c_s) that is predicted to encode b-1,3-glucan-binding protein (GNBP3) and GNBP1 (CL652Contig1), and pattern recognition receptors that recognize fungi and bacteria. Since the *E. intermedius* database was created from transcripts obtained from uninfected adult beetles and was thus not enriched for immune-related genes, the detection of these Toll pathway genes suggests that they were expressed constitutively in the adult beetle.

### Identity of the predicted serine proteases

Several proteases were identified by LCMS/MS analysis combined with sequence similarity searches using the MASCOT search engine as a first layer search on public databases without taxonomy restrictions. Furthermore, the sequences were manually compared to the *E. intermedius* database using TBLASTN. Using this approach, sequences of peptide fragments obtained from spots 2203 (molecular weight ≈30 kDa) and 3304 (molecular weight ≈50 kDa) matched proteins encoded by CL8Contig1, CL15Contig1, and CL20Contig1. These spots represented proteins that were up-regulated by fungal infection, and the matching transcripts encoded proteins that are predicted to be trypsin-like serine proteases. During immune response, serine proteases (SPs) and the non-catalytic serine protease homologues (SPHs), usually with N-terminal CLIP-domains, are known to mediate extracellular signaling activated by bacterial and fungal pathogens. SPHs are noncatalytic serine proteases where the catalytic triad has undergone mutations. Their function is poorly understood, but in some instances they have been reported to act as cofactors to the catalytic proteases (Gupta et al. 2005). In *H. diomphalia*, the prophenoloxidase (proPO) activating factor (PPAF-II) acts as a cofactor for PPAF-I in a limited proteolysis reaction to activate proPO (Kim et al. 2002).

**Figure 4.**
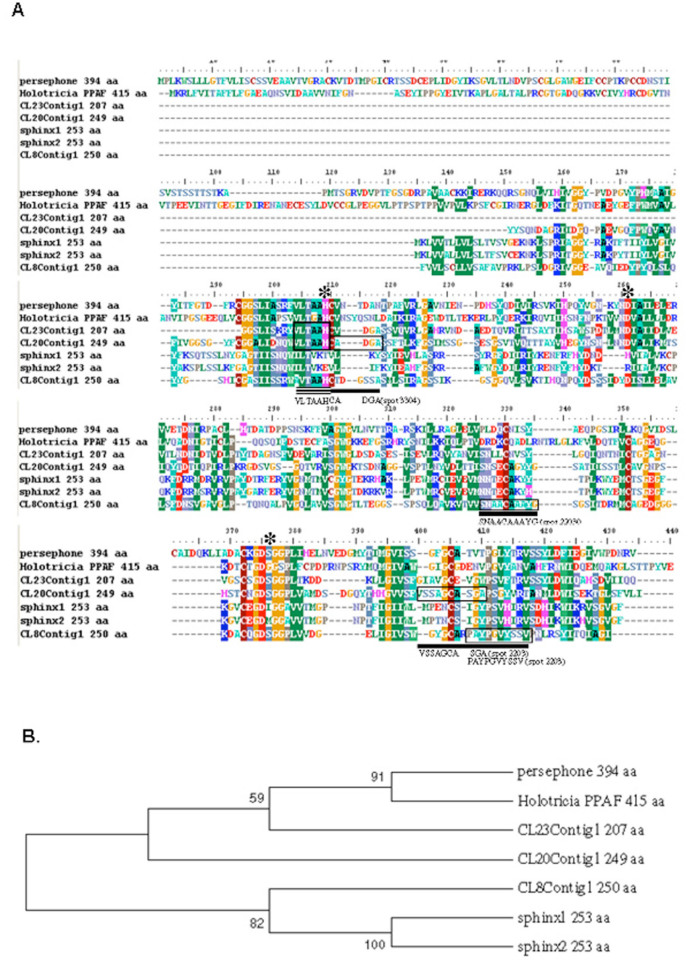
Sequence alignment of known proteases with predicted database polypeptides. A. *Drosophila* Persephone, sphinx proteins and *Holotricia diomphalia* PPAF were aligned with putative *Euonoticellus intermedius* SP domain proteins*.* The alignment was performed using the Clustal W program. The position of the catalytic triad is marked with asterisks. The triad is conserved in all three *E. intermedius* proteins. Peptide sequences obtained from the mass spectrometric analysis of the protein spots 2203 (single line) and 3304 (three lines) are aligned with one of the three *E. intermedius* sequences as shown. B. Phylogenetic tree of the abovementioned proteins*.* The tree was constructed using the neighbour joining method and the MEGA 5 software with 500 bootstrap replications. High quality figures are available online.

An alignment of protein sequences encoded by these contigs with Psh, *Holotricia* PPAF-II, and other clip-domain SPs and SPHs showed that the catalytic triad was conserved in all three *E. intermedius* predicted proteins, indicating that they may be SPs (Figure 4). The CLIP-domain is found in serine proteases such as Grass, Psh, and the SPH PPAF-II, but not in SPHs such as sphinx and spheroid (Kellenberger et al. 2011). The CLIP-domain SPs are known to be involved in the final steps of the proteolytic cascades. This study relied on the catalytic domains for analysis since the predicted *E. intermedius* proteins under investigation here did not have the full N-terminal sequence. Phylogenetic analysis conducted using the catalytic domain sequences showed potential relatedness between the *E. intermedius* proteins and those of other insects (Figure 4). In the neighbor-joining tree, *Drosophila* Psh and the *Holotricia* PPAF-II were grouped together with CL23Contig1 and possibly CL20Contig1, while sphinx1/2 may be related to CL8Contig1. Interestingly, *Tribolium* PPAF-II is predicted to be a Psh-like cofactor for putative proPO activating proteinases called TcSP7, SP8, or SP10 (Zou et al.2007). This may be evidence for the involvement of Psh-like proteases in the pro-PO pathway. It is notable that the catalytic triad was conserved in CL8Contig1 and mutated in sphinx, suggesting that CL8Conting1 belonged to the class of SPs.

### Other differentially expressed proteins in the haemolymph

In addition to the serine proteases, other proteins affected by fungal infection included pattern recognition receptor molecules, such as the apolipophorin III (spots 7203 and 7210 and CL123Contig1 in *E. intermedius* database) and the imaginal disc derived growth factor. Apolipophorin III and the imaginal disc derived growth factor protein spots are expressed at low levels in untreated beetles and elevated after fungal challenge. The Gram-negative-binding protein and the spaёtzle processing enzyme were detected by manually searching the *E. intermedius* database by TBLASTN using the *Drosophila* proteins. At least one Toll-like protein was predicted.

Peptide sequences obtained from spots 0101, 3004, 4002, and 9003 indicated possible matches with Pez when compared against the NCBI database. However, these sequences matched the odorant binding protein (OBP) family when compared to the *E. intermedius* database. The size range of these spots was consistent with that of OBPs and was too small for Pez. Protein spot 4002 appeared to be down-regulated, but statistical analysis indicated that the differences between protein expression in challenged and unchallenged beetles was not significant. Similarly, spot 9003 and 3004 appeared to be up-regulated, but statistical analysis indicated that the apparent differential expression was not significant. Protein in spot 0101 was, however, significantly up-regulated following fungal infection. The translated *E. intermedius* sequence (CL32Contig1) showed perfect matches with two alternative MS/MS peptide fragment sequences that were both identified as PBP/GOBP family when compared to the *E.intermedius* database (Figure 6). Interestingly, this transcript appeared to have two contiguous polyadenylation signals similar to those reported for human and horse growth hormones (Masuda et al. 1988; Ascacio-Martínez and Barrera-Saldaña 1994).

Spots 6410 and 5310 were predicted to be transferrins and gelsolin respectively. Other differentially expressed genes include the esterases and ATP synthase. Some of these proteins are usually associated with insect immune response, and others are known components of the haemolymph (Vierstraete et al. 2003; Karlsson et al. 2004).

## Discussion

Extracellular signal amplification and transduction during insect immune response is characterized by three categories of molecules that transduce the signal into the cell: the pattern recognition receptors, the SPs, and transmembrane receptors. Some components of the known extracellular signaling molecules were evident in *E. intermedius* based on proteomic analysis and the study of the adult transcriptome.

### Pattern recognition receptors

In insects, fungal pathogens are recognized by constitutively active and inducible pattern recognition receptors called GNBPs. Signals are transduced via an SP cascade in the extracellular space and across the membrane via the Toll receptor. In this study, GNBPs were not identified by proteomic analysis but were predicted in adult transcriptome of *E. intermedius* during manual searches of the database using sequences of *Drosophila* homologues. GNBPs and the related b-1,3-glucanrecognition proteins have been reported in several coleopterans, such as *Tribolium*. Evolutionarily b-1,3-glucan-recognition proteins are related to GNBPs and recognize bacterial and fungal cell wall components (Zou et al. 2007). Two inducible GNBP transcripts were identified in the burying dung beetle, *Nicrophorous vespilloides*, by suppression subtractive hybridization. These GNBPs cluster together with other coleopteran GNBPs from *Tribolium* (accession code NP_001164284) and *Tenebrio* (accession code BAG14263.1) with high bootstrap values (Vogel et al. 2011; Vogel et al. 2011).

Apolipophorin III was initially believed to be simply a subunit of lipid transport protein in the hemolymph (Pennington and Wells 2002). Later it emerged that apolipophorin III is important for insect immunity, and that it acts as a pattern recognition receptor that responds to b-1,3-glucan pathogen associated molecularpatterns found in fungi (Whitten et al. 2004; Rizwan-ul-Haq et al. 2011). Apolipophorin III has been shown to bind to bacterial and fungal cell wall components (Leon et al. 2006a, b) and is released into the haemolymph of insect larvae treated with lipopolysaccharide (Vogel et al. 2011). Here, the predicted *E. intermedius* apolipophorin III protein spots (molecular weight range 22–25 kDA) are significantly induced by fungal infection (Figure 2, Table 1). The precise effect of apolipophorin III interaction with pathogens is not well understood. It is reported, however, that apolipophorin III has a direct involvement with pathogens (Zdybicka-Barabas and Cytryńska 2011). The imaginal disc derived growth factor, also up-regulated by fungal infection (spot 7301), is a putative recognition protein with a chitin-binding lectin domain (Levy et al. 2004).

### Serine proteases

Invertebrates respond to bacterial or fungal infection by the activation of three pathways: the toll, the immune deficiency, and the proPO pathways. The toll pathway is activated by bacterial and fungal virulence factors via pattern recognition receptors such as GNBP3 and peptidoglycan recognition proteins. Alternatively, the toll pathway is activated by “danger signals” via a rather poorly-defined pathway characterized by an SP cascade involving Psh (Chamy et al. 2008). Psh is also implicated in the proPO pathway, as the *Manduca sexta* orthologous protein HP6 was found to induce proPO activation in plasma (An et al. 2009). These are distinct pathways at the extracellular level, but less so intracellularly, where signals are transduced via the NFkB and Relish transcription factors leading to expression of antimicrobial peptides. The activation of immune response by Grampositive bacteria and fungi is mediated by cascades of SP and results in the activation of spaёtzle, a cytokine-like molecule that acts as ligand for the transmembrane receptor Toll. Many of these proteases are synthesized as inactive precursor zymogens, which are cleaved by limited proteolysis to generate the downstream effector molecules. The proPO pathway leading to melanization is activated by peptidoglycan, b-1,3-glucan ,and by lipopolysaccharide via a protease cascade whose components are currently under investigation by many researchers (Lee et al. 1998; Jiang and Kanost 2000; Jiang et al. 2005; An et al. 2009). In this study, no clear evidence of molecules in this pathway was observed.

Activation by the major fungal cell wall component, b-1,3-glucan, is mediated via GNBP3, followed by a protease cascade that culminates in the activation of spaёtzle. A detailed study of this pathway in a coleopteran has been conducted on the larvae of *Tenebrio molitor* (Roh et al 2009), providing evidence for the existence of the Toll pathway activated by virulence factors. Earlier, the activation of the *Drosophila* Toll pathway by fungal and bacterial proteases (or so-called danger signals) was shown to be mediated by a parallel proteolytic cascade involving Psh (Chamy et al). The mechanism by which Psh is activated by fungal proteases and the identity of its substrate have so far not been clearly described. This pathway is known to be controlled by a serpin known as Necrotic, and all *necrotic* phenotypes are dependent on Psh, indicating a direct relationship between the two molecules (Ligoxygakis et al. 2002).

A clear homologue of Psh has not been identified in coleopterans. Based on the phylogenetic tree, the *E. intermedius* protein encoded by CL23Contig1 may be the candidate orthologue of Psh. Protein spots 2203 and 3304, whose intensity increases significantly after fungal infections, yielded several fragments that matched parts of the sequences of proteins encoded by CL8Contig1 and CL20Contig1. It is noteworthy that a putative Necrotic transcript (CL111Contig1) was detected in the *E. intermedius* database. Necrotic has been identified as a protease inhibitor that directly regulates the action of Psh. It has been reported that *nec* phenotypes are pleiotropic, including constitutive activation of spaёtzle and spontaneous melanization (Ligoxygakis et al. 2002). This reinforces the possible involvement of Psh in both the toll and proPO pathways. In *T. molitor*, three serpins that negatively regulate the Toll and proPO pathways were identified. These serpins are specific for each of the three apical SPs in the Toll pathway, and at least two of them are inducible by Lys-type peptidoglycan and b-1,3-glucan (Jiang et al. 2009).

Conceivably, the identity of these putative Psh transcripts can be confirmed by a combination of biochemical and genetic experiments involving *psh* and *nec* mutants. In *Tribolium*, 30 SPs and 18 SPHs were predicted, and a putative Psh orthologue named Tc-Sp66 was identified on the basis of induction by Grampositive bacteria and fungi (Zou et al. 2007). One orthologue was predicted to be Psh based on microarray evidence showing that its expression is induced only after fungal and Gram-positive bacterial challenge.

This study provides fair evidence for the presence of putative SPs that are up-regulated upon fungal infection. Better identification of these SPs requires cloning of the full-length transcripts as well as genetic and structural analyses of the genes and their respective proteins.

### Other proteins

In addition to the identification of peptides that match the putative pattern recognition receptors, trypsin-like SPs, and toll-like proteins, other proteins known to be involved in various aspects of host defense were putatively identified. These include transferrins, gelsolin, the OBP, and at least one protein tyrosine phosphatase. Transferrins are ironbinding proteins that regulate extracellular iron levels by sequestering iron atoms, thus preventing oxidative stress. They are particularly abundant in hematophagus insects such as Tsetse flies (Strickler-Dinglasan et al. 2006). Transferrin is often up-regulated after microbial infection and is part of the insect immune response, probably functioning as an antibiotic agent against pathogens (Lee et al. 2006).

Gelsolin is an actin-binding protein and is therefore a structural component of the cytoskeleton. It is found in two splice forms, cytoplasmic and secreted, in invertebrates such as *Drosophila* and is involved in cytoskeletal organization and biogenesis (Stella et al. 1994). It is involved in amyloid formation in vertebrates and haemolymph clotting in insects (Karlsson et al. 2004).

Insect OBPs are carrier proteins for transportation of small hydrophobic molecules through the haemolymph to receptors located in different tissues. They are small proteins (12–14 kDa) encoded by a divergent gene family. A comprehensive study showing diversity of these proteins and similarities among insects including coleoptera (*T. castaneum*) was conducted. This study showed emergence of subfamilies that probably arose through gene duplications (Gong et al. 2009). The existence of different isoforms that arose through gene duplications has been demonstrated in *T. molitor* (Graham et al. 2003). In *Tribolium*, remarkable expansion of odorant receptors was noted (Consortium 2008). Although OBP sequences differ significantly, they share high structural similarities, characterized by four conserved cysteines, which contribute to two disulfide bonds, a b-barrel and two a-helices (Rothemund et al. 1999; Graham et al. 2001; Graham and Davies 2002). There is some evidence of OBP involvement in insect immunity, but this is poorly defined (Levy et al. 2004). OBPs have been identified in normal haemolymph of *T. molitor* with no clear function except their potential to transport small hydrophobic molecules to tissues (Graham et al. 2003). In *Drosophila*, an OBP was found to be upregulated upon fungal infection and repressed by bacterial infection, suggesting an important role for these in immune response (Levy et al. 2004). Three protein spots were predicted to be differentially expressed OBPs, but only one of these was up-regulated upon fungal infection. The presence of multiple spots predicted to be OBPs suggests existence of different forms of the protein. The significance of the contiguous polyadenylation signals is currently not clear.

**Table 1.**
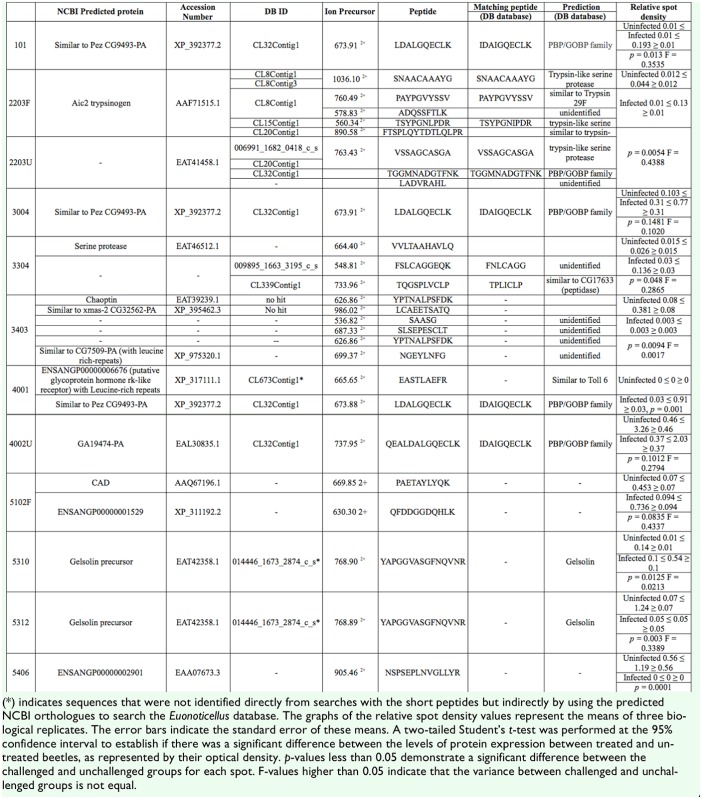
Up-regulated and down-regulated proteins.

**continued:**
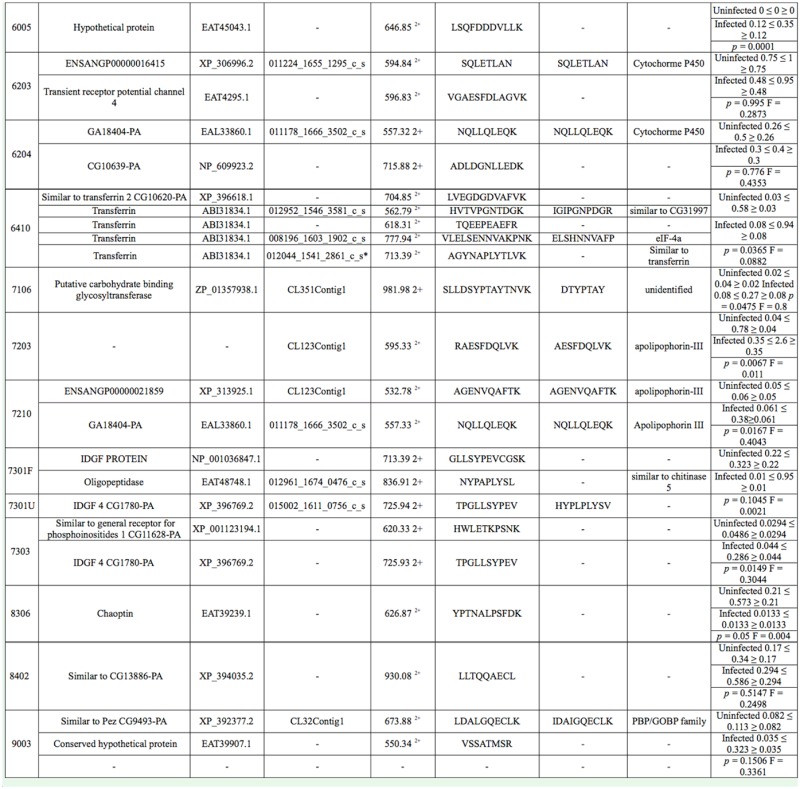

**Table 2.**
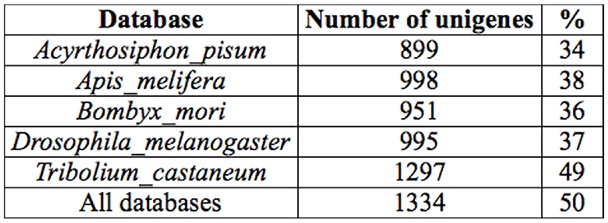
Comparison of the dung beetle database with those of other invertebrates. The total number of unigenes in the database was 2662.

**Table 3.**
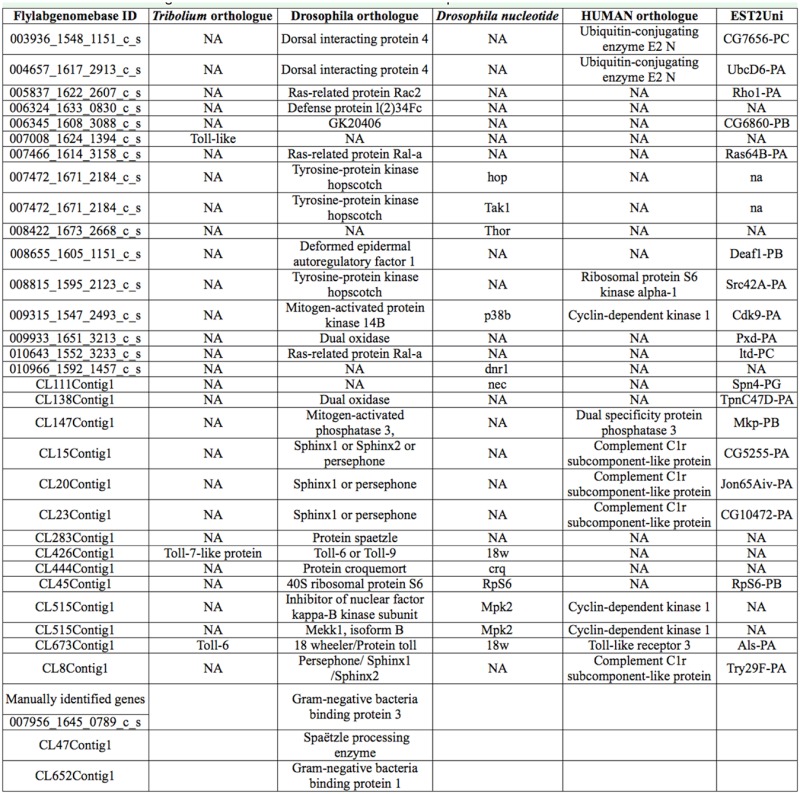
Predicted immune genes found in *Euonoticellus intermedius* transcriptome database.

**Figure 5.**
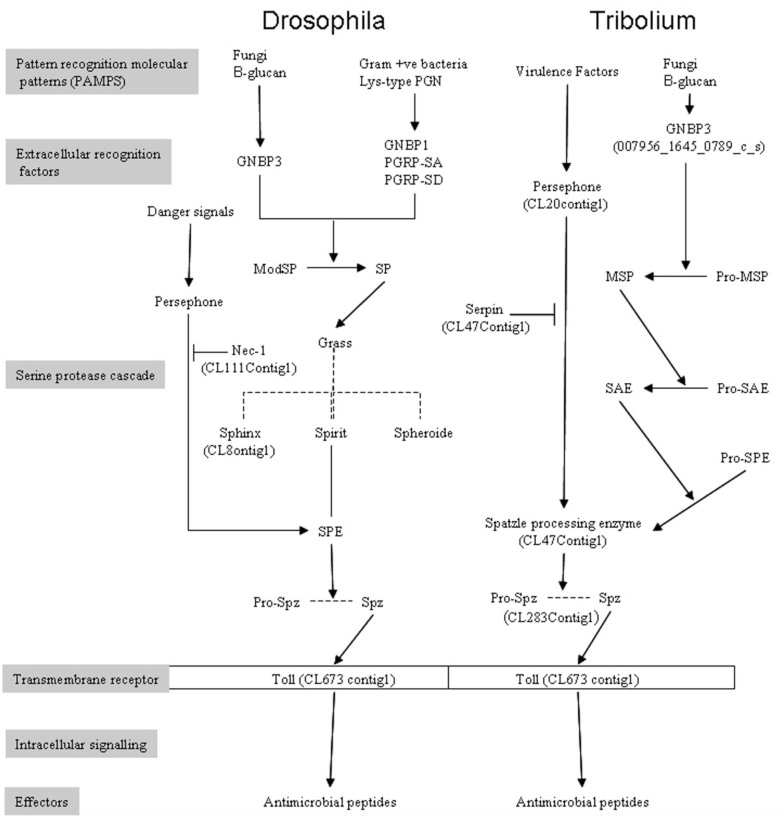
Schematic diagram of extracellular immune signaling in *Drosophila* and *Tribolium*. Pathogens are recognized by pattern recognition patterns (PRRs) based on pathogen associated molecular patterns (PAMPs) present on the pathogen. PAMPS may be Lys-type or DAP-type peptidoglycans. The pathway can also be activated by endogenous factors produced in live organisms (danger signals or virulence factors). The Toll pathway is activated when Gram-positive bacteria are sensed by the peptidoglycan recognition proteins (PGRPSA). Fungal PAMPS are sensed by the glucan binding protein 3 (GNBP3). These PRRs are secreted proteins, and when they form complexes with appropriate PAMPS, they initiate the serine protease cascades in the hemolymph, culminating in the cleavage of pro-spaёtzle, converting it to active spaёtzle, the endogenous ligand of the Toll receptor. In *Drosophila*, a parallel protease cascade is activated by danger signals such as fungal and bacterial proteases and activates the Toll pathway via the trypsin-like serine protease known as Persephone and spaёtzle. Binding of spaёtzle to Toll causes a conformational change and subsequent recruitment of intracellular signaling molecules. Intracellular signal transduction is mediated by a phosphorylation cascade that culminates in the release of NF-kB-like transcription factors from a complex with cactus. These transcription factors activate expression of genes that encode for antimicrobial peptides and other effectors. High quality figures are available online.

**Figure 6.**
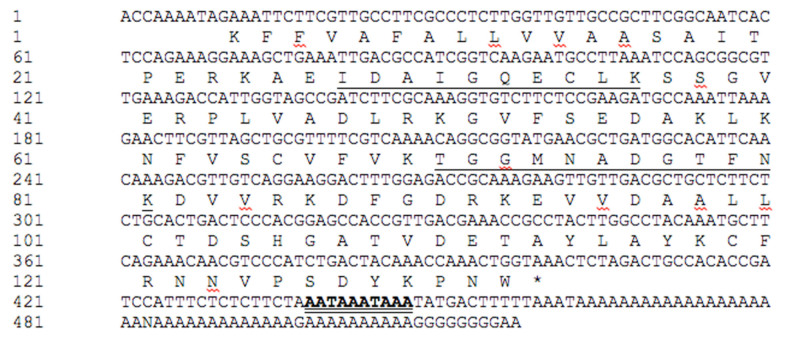
Translation of CL32Contig1. Peptide fragment sequenced obtained by MS/MS are underlined. Putative overlapping tandem polyadenylation signals in bold and double underline. High quality figures are available online.

## Abbreviations

**GNBP**,: Gram-negative binding protein;
**NCBI**,: United States National Center for Biotechnology Information;
**OBP**,: odorant binding protein;
**Psh**,: Persephone;
**proPO**,: prophenoloxidase;
**SPs**,: serine proteases;
**SPHs**,: serine protease homologues
